# Altered Lipid Metabolism Impairs Skeletal Muscle Force in Young Rats Submitted to a Short-Term High-Fat Diet

**DOI:** 10.3389/fphys.2018.01327

**Published:** 2018-09-26

**Authors:** David E. Andrich, Ya Ou, Lilya Melbouci, Jean-Philippe Leduc-Gaudet, Nickolas Auclair, Jocelyne Mercier, Blandine Secco, Luciane Magri Tomaz, Gilles Gouspillou, Gawiyou Danialou, Alain-Steve Comtois, David H. St-Pierre

**Affiliations:** ^1^Département des Sciences de l’Activités Physique, Université du Québec à Montréal, Montreal, QC, Canada; ^2^Groupe de Recherche en Activité Physique Adaptée, Université du Québec à Montréal, Montreal, QC, Canada; ^3^Département des Sciences Biologiques, Université du Québec à Montréal, Montreal, QC, Canada; ^4^Centre de Recherche du CHU Sainte-Justine, Montreal, QC, Canada; ^5^Centre de Recherche de l’Institut de Cardiologie et de Pneumologie de Québec, Ville de Québec, QC, Canada; ^6^Royal Military College Saint-Jean, Saint-Jean-sur-Richelieu, QC, Canada

**Keywords:** high-fat diet, young rats, muscle contractility, lipid droplets, muscle fibers

## Abstract

Obesity and ensuing disorders are increasingly prevalent in young populations. Prolonged exposure to high-fat diets (HFD) and excessive lipid accumulation were recently suggested to impair skeletal muscle functions in rodents. We aimed to determine the effects of a short-term HFD on skeletal muscle function in young rats. Young male Wistar rats (100–125 g) were fed HFD or a regular chow diet (RCD) for 14 days. Specific force, resistance to fatigue and recovery were tested in *extensor digitorum longus* (EDL; glycolytic) and soleus (SOL; oxidative) muscles using an *ex vivo* muscle contractility system. Muscle fiber typing and insulin signaling were analyzed while intramyocellular lipid droplets (LD) were characterized. Expression of key markers of lipid metabolism was also measured. Weight gain was similar for both groups. Specific force was decreased in SOL, but not in EDL of HFD rats. Muscle resistance to fatigue and force recovery were not altered in response to the diets. Similarly, muscle fiber type distribution and insulin signaling were not influenced by HFD. On the other hand, percent area and average size of intramyocellular LDs were significantly increased in the SOL of HFD rats. These effects were consistent with the increased expression of several mediators of lipid metabolism in the SOL muscle. A short-term HFD impairs specific force and alters lipid metabolism in SOL, but not EDL muscles of young rats. This indicates the importance of clarifying the early mechanisms through which lipid metabolism affects skeletal muscle functions in response to obesogenic diets in young populations.

## Introduction

There are growing concerns regarding the increased prevalence of obesity and ensuing metabolic disorders in young populations (Lobstein et al., 2004). Obese children are highly at risk of developing juvenile insulin resistance (Ong et al., 2004; Huang et al., 2011), type 2 diabetes (Pulgaron and Delamater, 2014) and other health conditions that will deteriorate in later stages of life (Reilly and Kelly, 2011). Poor diet quality and sedentarity are leading causes of childhood obesity (Emmett and Jones, 2015) and the excessive uptake of dietary fats amplifies this phenomenon (McGloin et al., 2002). Under obesogenic conditions, lipids accumulate in adipose tissue as well as in various tissues and organs, such as the liver and skeletal muscles (Tomlinson et al., 2016).

Long-term exposure to obesogenic diets was shown to cause atrophy in different leg muscles in middle-aged mice (Lee et al., 2015). This was also associated to a shift in skeletal muscle fiber type distribution. In mice, an obesogenic diet altered skeletal muscle fiber type distribution toward a glycolytic phenotype in the soleus (Denies et al., 2014) while the opposite effect was observed in the quadriceps (de Wilde et al., 2008). Hence, 12 weeks of HFD significantly changed the composition of fast-twitch fibers and decreased muscle force in adult mice (Eshima et al., 2017). Contractile properties of both fast and slow twitch muscles were also impaired in response to changes in fiber types after only 5 weeks of HFD in mice (Ciapaite et al., 2015). In rats, intramyocellular lipid accumulation and shifts in fiber type distribution occurred after 10 weeks of HFD (Kaneko et al., 2011). Interestingly, a significant increase in intramuscular lipids was noted in response to 2 days of HFD in the red portion of oxidative muscles and after 3–4 days in the white section of glycolytic muscles in young male rats (Bonen et al., 2015). Excessive intramuscular lipid accumulation is also proposed as an important mechanism promoting insulin resistance in the skeletal muscle (Putti et al., 2016). Altogether, this strongly suggests that the onset and development of intramyocellular lipid infiltration, fiber phenotype remodeling, alteration of contractile properties and insulin resistance follow different timelines in response to HFD in the skeletal muscle. It also appears that the rate at which the deleterious effects of HFD occur and progress is faster in a young population. This notion is paramount, since the early development of obesity-related dysfunctions in children and teenagers is likely to worsen in later stages of life (Guo and Chumlea, 1999; Harris et al., 2006). Hence, the impact of obesogenic diets on the alteration of skeletal muscle functions remains ill-defined in young populations submitted to obesogenic diets. Therefore, the present study intended to unravel the effects of a short-term (14 days) HFD on contractile properties, fiber phenotype, insulin signaling and lipid metabolism in oxidative (soleus; SOL) and glycolytic (*extensor digitorum longus*: EDL) skeletal muscles (Delp and Duan, 1996) of young rats. Thus, we hypothesized that a short-term exposure to an obesogenic diet promotes intramyocellular lipid accumulation and insulin resistance while decreasing force production and altering fiber type distribution in SOL and EDL muscles.

## Materials and Methods

### Animal Procedures

This study was carried out in strict accordance with recommendations of the National Institutes of Health guide for the care and use of Laboratory animals. Before undergoing the experimental work, the protocol was approved by the *Comité Institutionnel de Protection des Animaux* (CIPA) of UQAM (Permit Number: 0515-R3-759-0516). After a 3-day acclimatization period at UQAM’s animal facility, young (100–125 g; ∼4 weeks old) male Wistar rats (Charles River, Canada) were randomly allocated to regular chow (RCD; *n* = 8) or HFD (*n* = 8) diets for 14 days. Animals were given free access to food and water and submitted to a 12-h light/dark cycle (6 to 18 h). Rats were individually weighed daily at the same time-period. Sacrifices were performed after a 4 h fast to standardize the feeding status of each animal. In different sets of experiments, skeletal muscles (SOL and EDL) from both legs were collected for assessment of fiber phenotyping, intramyocellular lipid characterization, insulin signaling and gene expression. For the muscle contractility experiments, EDL and SOL muscles were surgically harvested from the left leg for each animal.

### Diets

The high fat diet (HFD) was prepared from purified food-grade reagents according to a commercial formulation (D12492 diet, Research Diets Inc., New Brunswick, NJ, United States) as previously reported (Andrich et al., 2018). It had a physiological fuel value (calculated using a modified Atwater factor) of 20.09 kJ/g (4.80 kcal/g) and a macronutrient weight content of 26.3% carbohydrate (19.2% kJl), 26.2% protein (19% kJ) and 34.9% fat (61.8% kJ). The regular chow diet (Charles River Rodent Diet # 5075, Cargill Animal Nutrition, United States) had a physiological fuel value of 12.09 kJ/g (2.89 kcal/g) and a macronutrient weight content of 55.2% carbohydrate (65.6% kJ), 18% protein (21.4% kJ) and 4.5% fat (13% kJ).

### Muscle Contractility

The contractility apparatus (1205A, Aurora Scientific, Aurora, ON, Canada) was adjusted before each experiment using calibration weights (10, 20, and 50 g). Rats were anesthetized with isoflurane (induction: 4% and maintenance: 2%) at 1 l/min of O_2_. The *ex vivo* skeletal muscle contractility protocol was adapted from Lynch et al. (2001). A surgical silk suture (size 3.0) was tied to each tendon while preserving the muscle’s integrity. The muscle was mounted between two platinum electrodes in a muscle bath containing 50 ml of Krebs solution that was maintained at a temperature of 30°C and constantly bubbled with 95% O_2_ and 5% CO_2_ and where, using the silk threads, one end of the muscle tendon was attached to the apparatus and the other end was attached to the servomotor. After a 15 min thermal equilibration period, the length at which maximal force (g) is produced was determined by gradual length adjustments while submitting the muscle to 5 Hz stimulations. Once this optimal length (L_0_) was obtained, it was continuously used throughout the experiments. After 15 min of rest, the contractility protocol was initiated by stimulating the muscle at 5, 10, 30, 50, 75, 100, 120, and 150 Hz for 600 ms at each frequency with a 2 min pause between each contraction. After this force-frequency protocol, muscles were rested for 3 min. The fatigue protocol was then initiated by stimulating the EDL (50 Hz) and SOL (75 Hz) muscles every 2 s over a 3 min period. The time necessary to decrease the initial force by 50% was defined as fatigue resistance. After the end of the fatigue period, muscles rested for 3 min before undergoing the recovery protocol. This was performed by submitting the EDL (50 Hz) and SOL (75 Hz) to stimulations every 90 s over a period of 30 min. All variables were calculated using the DMC software (Aurora Scientific, Aurora, ON, Canada) by measuring force, the time between the initiation of the contraction and the maximal force produced (time to maximum) as well as the time necessary to reach 50% of the maximal force (half relaxation time). Once stimulations were completed muscles were carefully cleaned, dried and weighed. Specific force (N/cm^2^) was obtained by dividing the muscle force (g) and gravitational acceleration (9.81 m/s^2^) product by the estimated muscle cross-sectional area (CSA). The latter was calculated by dividing the muscle’s weight (g) by the product of the muscle density (1.06 mg/mm^-3^) and muscle length (L_0_).

### Muscle Fiber Phenotype

To categorize muscle fibers, EDL and SOL muscles (right leg) were collected and cleaned of all adipose and connective tissues. A slice of each muscle’s entire middle belly was mounted in tragacanth gum on a cork disk, frozen in liquid nitrogen-cooled isopentane and stored at -80°C until further use. Muscle cross-sections (8 μm) were cut using a CM1950 cryostat (Leica Biosystems, Wetzlar, Germany), at -18°C and mounted on lysine slides (Superfrost, Thermo Fisher Scientific). Sections were then immunolabeled for the different myosin heavy chains (MHC) as previously described (Goodman et al., 2011). Cross-sections of each muscle sample were immunolabeled for MHC type I, IIa, IIb (cocktail 1), or IIx (cocktail 2), left to reach room temperature, rehydrated with PBS (pH 7.2), blocked using goat serum (10% in PBS) and incubated with the primary antibody cocktail for 1 h. Cross-sections were then washed (3 ×) with PBS, incubated with the secondary antibody cocktail for 1 h, washed (3 ×) with PBS and cover slipped with an antifade liquid mountant (ProLong Gold, Invitrogen, P36930). Primary and secondary antibodies as well as their respective concentration were used as described earlier (Gouspillou et al., 2014). Primary antibodies used for cocktail 1 were: mouse IgG2b monoclonal anti-MHC type I (BA-F8, 1:25), mouse IgG1 monoclonal anti-MHC type IIa (SC-71, 1:200), mouse IgM monoclonal anti-MHC type IIb (BF-F3, 1:200), and rabbit IgG polyclonal anti-laminin (Sigma L9393, 1:750). Primary antibodies used for cocktail 2 were: mouse IgM monoclonal anti-type IIx MHC (6H1, 1:25) and rabbit IgG polyclonal anti-laminin. Secondary antibodies used for cocktail 1 were: Alexa Fluor 350 IgG2b (y2b) goat anti-mouse (Invitrogen, A-21140, 1:500), Alexa Fluor 594 IgG1 (y1) goat anti-mouse (Invitrogen, A-21125, 1:100), Alexa Fluor 488 IgM goat anti-mouse (Invitrogen, A-21042, 1:500) and Alexa Fluor 488 IgG goat anti-rabbit (A-11008, 1:500). Secondary antibodies used for cocktail 2 were: Alexa Fluor 488 IgM goat anti-mouse and Alexa Fluor 488 IgG goat anti-rabbit. All primary antibodies targeting MHCs were purchased from the Developmental Studies Hybridoma Bank (DSHB, University of Iowa, IA, United States).

### Western Blotting

Insulin receptor signaling was measured following an insulin challenge in rats. After a 6 h fast (from 6 to 12 h), rats were anesthetised and administered with 38 μl/kg insulin (Humulin, Lilly, Canada). After 5 min, SOL and EDL muscles were quickly collected and weighed as previously described (Caron et al., 2017). Briefly, muscle tissues were homogenized in a buffer (50 mM HEPES, pH 7.4, 2 mM EDTA, 10 mM sodium pyrophosphate, 10 mM sodium glycerophosphate, 40 mM NaCl, 50 mM NaF, 2 mM sodium orthovanadate, 1% Triton-X 100, with one tablet of Roche EDTA-free protease inhibitor per 25 ml solution) supplemented with 1% sodium deoxycholate and 0.1% sodium lauryl sulfate. Proteins were extracted and Bradford assays were performed to quantify protein levels. Samples were loaded on 4–12% NuPAGE precast gels or 10% Tris-Glycine Gels (Life Technologies). Proteins were transferred to PVDF membranes blocked in 5% milk diluted in PBS-Tween and incubated overnight with their primary antibody at 4°C. The following antibodies were used: IRS-1 (Cell Signaling Technology, 2382, dilution 1:1000), Akt (Cell Signaling Technology, 4691, dilution 1:1000), phospho-AKT S473 (Cell Signaling Technology, 9271, dilution 1:1000), S6 (Cell Signaling Technology, 2217, dilution 1:2500), phospho-S6 S240/244 (Cell Signaling Technology, 5364, dilution 1:1000) and secondary antibodies were purchased from Cell Signaling Technology (7076S and 7074S) and diluted 1:5000. Amersham ECL Western Blotting Detection Reagent (RPN2106) was used for imaging.

### Lipid Content

To characterize intramyocellular lipid content in muscles a fluorescent marker of neutral lipids, HCS LipidTOX Green Neutral Lipid Stain (Thermo Fisher, H34475) was used. Muscle cross-sections were fixed in 4% paraformaldehyde (PFA; Sigma-Aldrich, P6148) for 20 min then washed three times in PBS before being incubated with 0.275 μl/ml DAPI in PBS solution (Thermo Fisher, D3571) for 5 min. After three additional PBS washes samples were incubated for 30 min in a solution containing 1 μl/ml of HCS LipidTOX in PBS. After three further washes with PBS, cross-sections were cover slipped with DABCO (100 mg/ml PBS/ml glycerol with added sodium azide). Imaging was promptly performed.

### Slide Imaging and Image Analysis

Sections were imaged on a Nikon TE300 Microscope (Nikon Corporation, Tokyo, Japan) at UQAM’s imaging facility. Frames for analysis using the ImageJ software (NIH, United States) were randomly sampled across each muscle section. For muscle fiber type distribution, 400 individual fibers were traced on average. Fiber cross-sectional area and MHC type(s) were determined for each individual fiber. For lipid content, an average of 170 fibers were analyzed per cross-section. The number of lipid droplet (LD) per muscle fiber, % of fiber area covered by lipids and LD cross-sectional area (CSA) were measured. Lipid droplets with a CSA larger than 0.2 μm^2^ were considered as large lipid droplets.

### RT-qPCR

Samples were incubated in RNAlater (Ambion) and stored at -20°C for later use. Muscles (15–60 mg) were homogenized in 1 ml of TRIzol Reagent (Ambion) with a TissueLyser II homogenizer (QIAGEN) and extracted according to the manufacturer’s instructions. Samples were further processed using the PureLink RNA Mini Kit (Ambion) and contaminating DNA was removed via DNase on-column digestion. RNA concentrations and purity were determined by the ratio of absorbance at 260 and 280 nm (BioDrop). RNA integrity was evaluated by visualization of intact 18S and 28S RNA bands following agarose gel electrophoresis. SuperScript VILO Master Mix (Invitrogen) was used to synthesize cDNA with 1 μg of RNA per 20 μl reaction. Real-time PCR reactions were performed in triplicate using 384-well plates in the QuantStudio 6 system (Life Technologies) with SYBR Select Master Mix kit (Applied Biosystems). Reaction conditions consisted of 2 μl of a 1:10 cDNA dilution and 0.3 μM primers in a final volume of 10 μl. The following cycling protocol was used: 2 min at 50°C, 2 min at 95°C followed by 40 cycles of 15 s at 95°C, 15 s at 58°C, and 1 min at 72°C. The reaction’s specificity was verified with a melting curve analysis. Negative RT controls and no template controls (NTC) were included in the PCR runs. Reference genes were identified by running a TaqMan Array Rat Endogenous Control Plates (Applied Biosystems) with 3 samples for each group. Out of the 22 genes, the four with the best gene expression stability (Expression Suite, Applied Biosystems) were selected for normalization: PUM1, PGK1, PSMC4, and RPL30 for the EDL and PGK1, RPL30, PPIB, and PUM1 for the SOL. Primers used (**Table 1**) were designed using Primer-BLAST (NCBI). A standard curve obtained by scalar dilution of a cDNA pool was generated to verify PCR efficiency. Normalized and relative amounts of mRNA were calculated using the qbase+ (Biogazelle) software and presented as the fold change of the HFD group compared to the RCD group using the ΔΔCT method.

**Table 1:** Primers used for quantitative real-time PCR.

Gene	Forward/reverse primers
ACACA	GAAGTGACTGACTCCAGGACAG
	GGTCTTTGGTCACATACGGAGT
ACSS2	ACTTGGCGACAAAGTTGCTTTT
	TCTGAACACCCTGTTTACGGAG
ACTN3	TTCAACCACTTTGACCGGAAGC
	TGGTCATGATCCGAGCAAACTC
CEBPA	GCCAGCTAGAAAAATAGAAGCACT
	CATTTCTGCTTTCTCATCGCCG
CD36	GCCAAGAAGTCGGTGGATAAGA
	TTGTCACTGGTCAACTCCAACA
CEBPB	CTGAGCGACGAGTACAAGATGC
	CTTCTTCTGCAGCCGCTCGTTC
DGAT1	ACATTTCAGATTGAGAAGCGCC
	GGAACCCACTGGAGTGATAGAC
DGAT2	CCAAGAAAGGTGGCAGGAGAT
	CCATGGGGATGGTATCCAAAGA
FABP4	CCCCAGATGACAGGAAAGTGAA
	GCCTTTCATGACACATTCCACC
FASN	CTGGCCATGGTTTTAAGGGATG
	GAGAAGGCCACAAAGTAGTCCA
GLUT4	TGGTCTCGGTGCTCTTAGTAGA
	ATAACTCATGGATGGAACCCGC
LPL	GAAAGCCGGAGAGACTCAGAAA
	TGTTGGTTTGTCCAGTGTCAGC
MYL1	ACTCCAATGGCTGCATCAACTA
	CAAGCTGGTGTTGACAGTTAGC
MYOG	GCCGGTGGTACCCAGTGAA
	GATGGACGTAAGGGAGTGCAG
PGK1	AACAAGCTGACTTTGGACAAGC
	AGCAGCCTTGATCCTTTGGTTA
PPARG	GAAGAGCCTTCAAACTCCCTCA
	GCTTCAATCGGATGGTTCTTCG
PPARGC1A	AGAGTCACCAAATGACCCCAAG
	TTGGCTTTATGAGGAGGAGTCG
PPIB	ACAGGAGAGAAAGGATTTGGCTA
	CGCTCACCATAGATGCTCTTTC
PSMC4	GCTCTACAAGCAGATTGGCATC
	TCTGAACAAACTCTGAGCCCAC
PUM1	GAACACCAGGTGCGCTCTAT
	CCTTTTGGTCCATCTTTGCTGG
RPL30	TGGCTGCAAAGAAGACGAAAAA
	AACCAATTTCGCTTTGCCTTGT
SLC27A4	CACTCAGCAGGAAACATTGTGG
	GGCAAAGCTCACCAATGTACTG


### Statistical Analyses

Sample sizes were calculated as recommended (Charan and Kantharia, 2013) using data from previously published studies as well as our own pilot studies using power set at 0.8 (80%) and significance set at *P* < 0.05. All values are presented as means ± SD, except where specified. Distributions’ normality was assessed using the Shapiro-Wilk test. Two-tailed unpaired Student’s *t-*tests were used to compare values between the two groups. Mann-Whitney U tests were used to compare values between both groups when data did not meet assumptions of parametric testing. Statistical analyses were performed using the SPSS 16.0 (IBM Corporation, Armonk, NY, United States) and SAS Studio 3.5 (SAS Institute, Cary, NC, United States) softwares.

## Results

**FIGURE 1:**
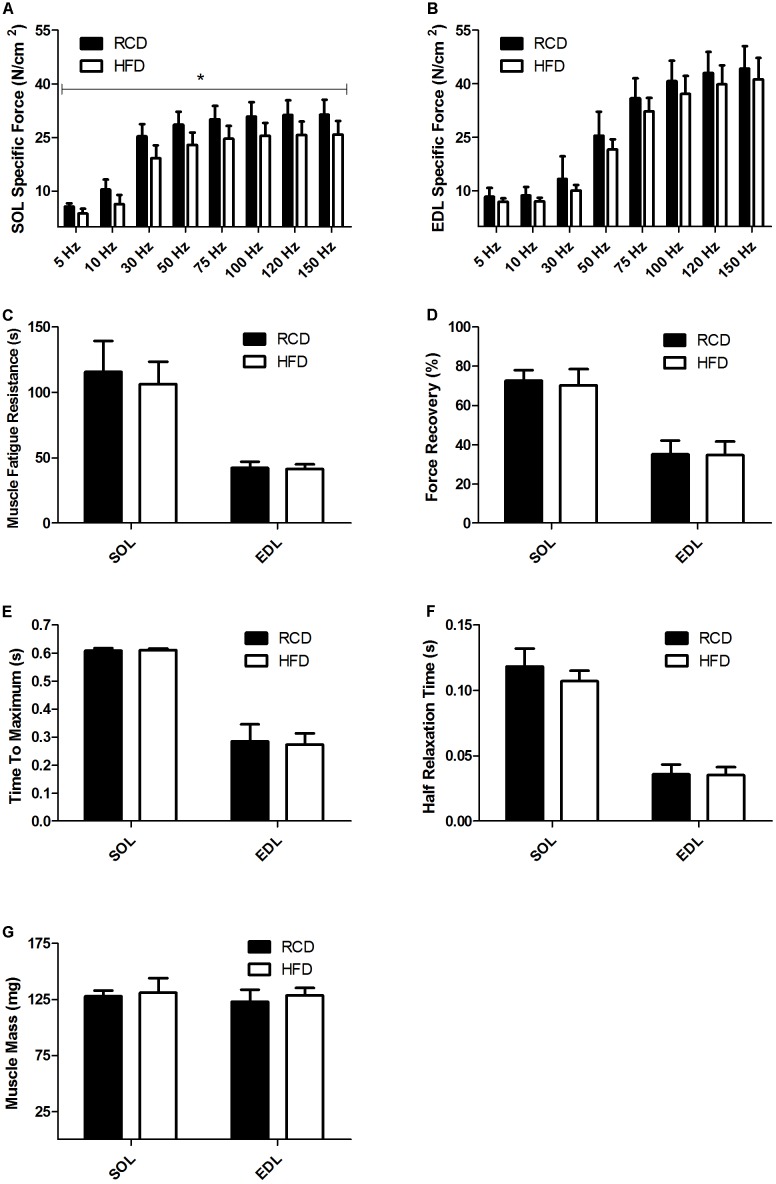
Skeletal muscle specific force of SOL **(A)** and EDL **(B)** muscles of young rats submitted to HFD or RCD for 14 days. Fatigue resistance **(C)**, force recovery **(D)**, time to maximum contraction **(E)**, half relaxation time **(F)**, and muscle mass **(G)** of the SOL and EDL muscles are shown. Unpaired Student’s *t*-tests were used to compare values between both groups. Results are presented as means ± SD for *n* = 8; ^∗^indicates significant difference between the two groups (*P* < 0.05).

As previously reported by our group, no difference in body weight was noted between HFD and RCD rats (Andrich et al., 2018). Further, the muscle weight and CSA of the EDL and SOL muscles were similar in both groups (**Table 2**). Contractility tests showed a significant decrease in specific force production for the SOL muscle of rats submitted to HFD (*P* = 0.014; **Figure 1A**). Force production of the EDL was similar for both groups (*P* = 0.125; **Figure 1B**). The obesogenic diet did not induce any difference in resistance to fatigue (**Figure 1C**), muscle recovery to fatigue (**Figure 1D**), maximum contraction (**Figure 1E**) and half relaxation time (**Figure 1F**) for EDL and SOL muscles in young rats. This indicates that contraction and relaxation rates were unaltered by HFD and both oxidative and glycolytic muscles maintained their distinct contraction profiles. The mass of EDL and SOL muscles was also similar in both groups (**Figure 1G**).

**Table 2:** Biometric parameters of RCD and HFD rats.

	RCD	HFD	*p*
Body Weight Day 1 (g)	152.69 ± 19.33	156.77 ± 18.79	0.686
Body Weight Day 14 (g)	295.03 ± 25.02	290.60 ± 12.04	0.666
Body Weight Gain (g)	142.34 ± 14.94	133.83 ± 11.27	0.241
SOL Weight (mg)	128.00 ± 4.81	131.15 ± 12.88	0.533
EDL Weight (mg)	123.11 ± 10.59	128.54 ± 6.72	0.242
SOL CSA (mm^2^)	4.49 ± 0.43	4.95 ± 5.90	0.102
EDL CSA (mm^2^)	3.89 ± 0.58	4.39 ± 0.45	0.079


*Means ± SD. RCD, regular chow diet; HFD, high fat diet.*

**FIGURE 2:**
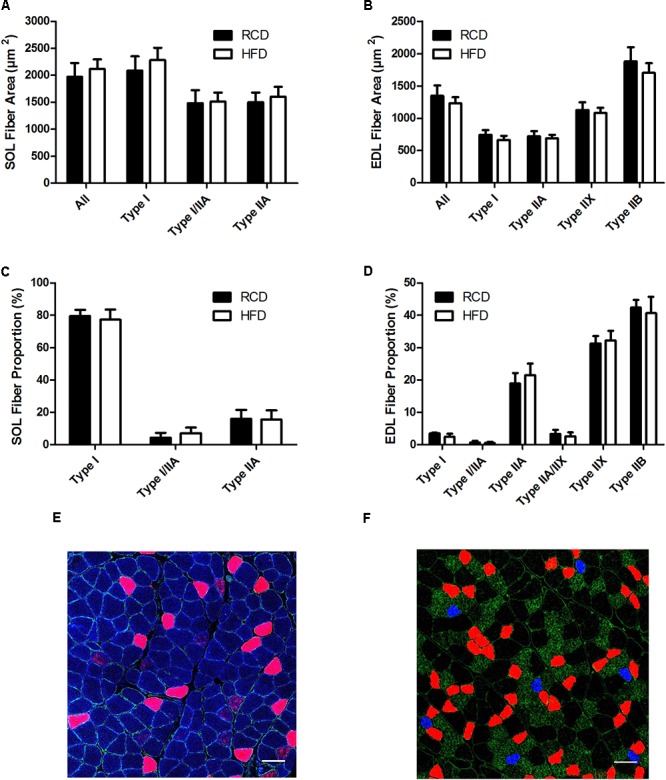
Cross-sectional areas of different fiber types in the SOL **(A)** and EDL **(B)** muscles of young rats submitted to HFD or RCD for 14 days. Fiber type proportions in the SOL **(C)** and EDL **(D)** muscles are shown. *In situ* immunolabeling of a SOL **(E)** and EDL **(F)** muscle cross-section for myosin heavy chains type I (blue), IIa (red), IIb (green), and IIx (black) are shown. Unpaired Student’s *t*-tests were used to compare values between both groups. Results are presented as means ± SD for *n* = 6–7. Scale bar: 100 μm.

Areas (**Figures 2A,B**) and the proportion (**Figures 2C–F**) of different fiber types were similar for both SOL and EDL muscles in HFD and RCD groups, indicating that decrease in specific force observed in SOL is not due to a shift in muscle fiber type distribution.

**FIGURE 3:**
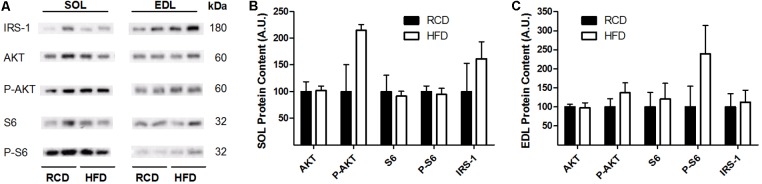
Western blot showing the expression of insulin signaling proteins in oxidative (SOL) and glycolytic (EDL) skeletal muscles of young rats submitted to 14 days of HFD or RCD **(A)**. Blots were cropped for display. Quantification of IRS-1, Total Akt, phospho-Akt, Total S6, and phospho-S6 protein contents (normalized for their corresponding ponceau stain intensities) are shown in SOL **(B)** and EDL **(C)** muscles. Mann-Whitney U tests were used to compare values between both groups. Results are presented as means ± SEM for *n* = 4.

In response to the diets, levels of insulin receptor signaling mediators IRS-1, AKT, phospho-AKT, S6 and phospho-S6 were not modulated in the EDL or SOL muscles (**Figures 3A–C**; **Supplementary Figure S1**). This indicates that insulin receptor signaling was unaffected by the obesogenic diet after 2 weeks.

**FIGURE 4:**
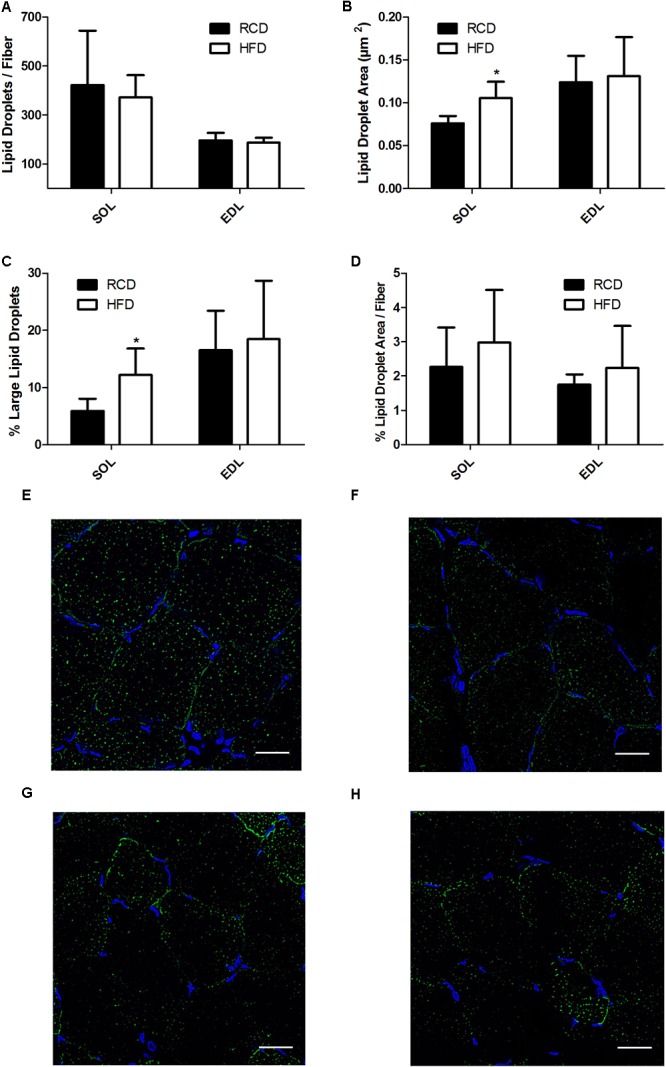
Number of lipid droplets per fiber of SOL and EDL muscles of young rats submitted to HFD or RCD for 14 days **(A)**. Average droplet cross-sectional area **(B)**, percentage of large droplets **(C)** as well as percentage of fiber area covered by lipid droplets **(D)** in SOL and EDL muscles are shown. *In situ* fluorescent staining of lipid droplets in SOL muscles of HFD **(E)** and RCD **(F)** rats as well as in EDL muscles of HFD **(G)** and RCD **(H)** rats. Unpaired Student’s *t*-tests were used to compare values between both groups. Results are presented as means ± SD for *n* = 5–7; ^∗^indicates significant difference between the two groups (*P* < 0.05). Scale bar: 20 μm.

In HFD rats, average LD numbers per muscle fiber were not changed in SOL or EDL muscles (**Figure 4A**). However, the average size of LDs (cross-sectional area; CSA) was significantly larger in SOL muscles of HFD rats (*P* = 0.015; **Figures 4B,E–H**) but this effect was not detected in EDL muscles. Further, large LDs were found in higher proportion in SOL (*P* = 0.024; **Figure 4C**) but not EDL (*P* = 0.024; **Figure 4C**) muscles of HFD rats. Percent LD area per fiber was similar in SOL and EDL muscles for both treatment groups (**Figure 4D**). Overall, these results suggest that larger LDs impair force production mechanisms in SOL muscles of rats treated with HFD.

**FIGURE 5:**
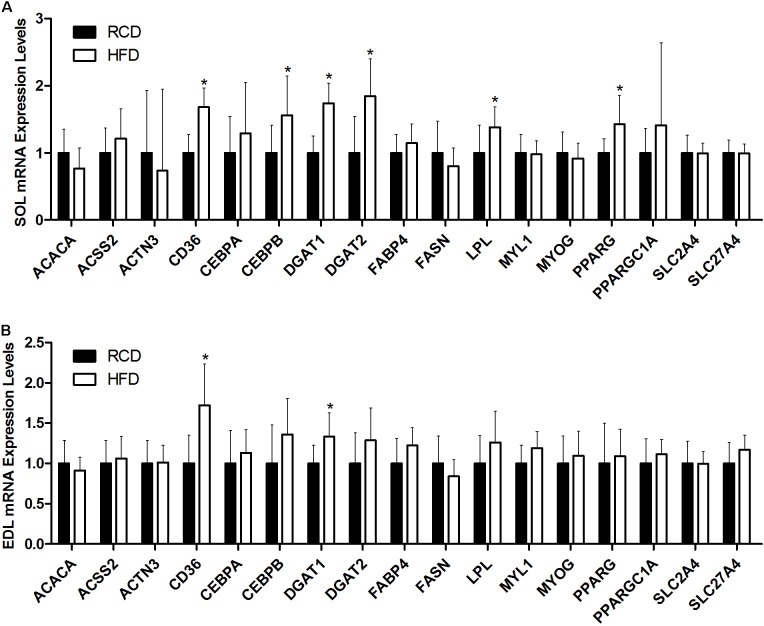
Expression levels of genes implicated in lipid and muscle metabolisms in the SOL **(A)** and EDL **(B)** muscles of young rats submitted to HFD or RCD for 14 days are shown. Results are presented as mean fold change ± SD of the HFD group compared to the RCD group using the ΔΔCT method for *n* = 15; ^∗^indicates significant difference between the two groups (*P* < 0.05).

In SOL muscles (**Figure 5A**), the expression of CD36 (∼68%; *P* < 0.001), CEBPB (∼56%; *P* = 0.006), DGAT1 (∼74%; *P* < 0.001), DGAT2 (∼84%; *P* = 0.002), LPL (∼38%; *P* = 0.020), and PPARG (∼43%; *P* = 0.002) genes was significantly increased in HFD rats. In turn, the expression of CD36 (∼72%; *P* < 0.001) and DGAT1 (∼33%; *P* = 0.004) was also higher in EDL muscles of the HFD group (**Figure 5B**). This indicates that the transcription of genes involved in adipogenic maturation (PPARγ and C/EBPβ), intracellular lipid uptake (CD36), triacylglycerol synthesis (DGAT1 and DGAT2) and extracellular lipolysis (LPL) is upregulated in response to a short-term HFD in SOL muscles.

## Discussion

The present study intended to clarify the effects of a short-term 14 days HFD on a host of functions in oxidative and glycolytic skeletal muscles. The major findings are that, in response to the short-term HFD, oxidative SOL muscles produced significantly less specific force and had larger intramyocellular LDs. Further, the expression of key mediators of lipid metabolism involved in adipogenic maturation, intracellular lipid uptake, triacylglycerol synthesis and extracellular lipolysis is upregulated in SOL muscles of HFD rats. However, only the expression of CD36 and DGAT1 was upregulated in the EDL. Consequently, it appears that modifications in the intrinsic nature of LDs impair the contractile force in a skeletal muscle with oxidative properties. It is noteworthy that these effects even precede alterations in skeletal muscle fiber type distribution and insulin receptor signaling. Thus, to our knowledge and relevant to the originality of the present work, no other studies integrating mechanistic and functional muscle physiology, such as specific force, resistance to fatigue and recovery, fiber phenotyping, insulin receptor signaling, LD characterization and the expression of several mediators of lipid metabolism in young rats subjected to a HFD for only 14 days have been identified.

The present results show that skeletal muscle fiber type distribution is not affected by a short-term HFD in young rats. This was primarily investigated since maximal specific force is largely dependent of fiber type distribution (Eddinger and Moss, 1987; Ranatunga and Thomas, 1990) and myosin heavy chain content in the skeletal muscle (Geiger et al., 2000). It was previously suggested that obesogenic diets decrease force by inducing a remodeling of skeletal muscle fibers (Ciapaite et al., 2015; Lee et al., 2015; Eshima et al., 2017). However, our results are supported by the fact that impairments in SOL specific force were not associated with changes in fiber typing in mice submitted to 5 weeks of HFD (Ciapaite et al., 2015). Interestingly, the same group observed changes in lipid composition and mitochondrial functions in EDL, but not in SOL. Our results show that larger LDs were observed in SOL, but not in EDL muscles; these discrepancies could be explained by differences in the experimental model, namely by the age and the species tested. Further, we have recently reported that 14 days of HFD significantly increase palmitic acid utilization while altering the expression of genes involved in the regulation of mitochondrial dynamics in both oxidative and glycolytic muscles of young rats (Leduc-Gaudet et al., 2018). Interestingly our results obtained with the same model show a decline in ambulatory activity in response to the short-term HFD (Andrich et al., 2018). This suggests that in response to obesogenic diets, skeletal muscle impairments could play a role in the etiology of sedentary behaviors in young rats. Otherwise, impairments in specific skeletal muscle force have been mainly observed after submitting rodents to HFD for longer time periods (Lee et al., 2015; Eshima et al., 2017). On the other hand, the role of intramyocellular lipids in muscular force was previously suggested in young and elderly human subjects, in which force production of the vastus lateralis negatively correlated with intramuscular lipids measured by MRI (Hioki et al., 2016). Similarly, using biopsies from elderly obese or normal-weight participants, altered contractile functions were observed in single muscle fibers containing high levels of intramyocellular lipids (Choi et al., 2016). It is noteworthy that other co-factors should be considered when investigating skeletal muscle functions in elderly populations. Indeed, collagen accumulations decrease muscle elasticity (Kragstrup et al., 2011) and motor unit array compression (Yoshida et al., 2012) and these factors are suggested to be main contributors promoting impairments in muscle quality in aged individuals. Such age-related alterations of skeletal muscle parameters can be excluded in the present study. However, skeletal muscle alterations would likely be amplified in aged rats chronically submitted to HFD. Hence, this emphasizes the important role of enhanced intramyocellular lipid accumulation in the impairment of force production in SOL muscles of young individuals.

Another potential mechanism through which muscular force can be impaired involves delayed myoplasmic calcium clearance (Summermatter et al., 2012). This effect was observed in response to PGC1α overexpression in mice and was associated with reduced maximal force, increased resistance to fatigue and fiber type remodeling in the EDL muscle. The overexpression of PGC1α also promoted a fiber-type switch from a glycolytic toward a more oxidative phenotype in mice (Lin et al., 2002). In line with our previous work (Leduc-Gaudet et al., 2018), we could not observe any difference in the expression of PGC1α, suggesting that mitochondrial biogenesis is not altered in SOL or EDL muscles in response to our short-term HFD. Overexpression of PPARγ was also observed in the SOL muscle. This factor is a key player in adipocyte differentiation and metabolism; it has been suggested as a promoter of insulin resistance under a high fat diet via its role in adipocyte hypertrophy (Kubota et al., 1999), although its role differs in skeletal muscle (Kintscher and Law, 2005). The expression of C/EBPβ was also positively modulated in SOL muscles of HFD rats. However, the increased C/EBPβ expression in leukocyte-derived RNA samples were previously associated with increased muscle strength in humans (Harries et al., 2012) and enhanced satellite self-renewal leading to muscle regeneration in mice (Lala-Tabbert et al., 2016). Conversely, C/EBPβ is also involved in the process through which pre-adipocytes are differentiated toward a mature adipocyte phenotype (Tanaka et al., 1997). Thus, it is appealing to postulate that the modulatory effect of C/EBPβ on skeletal muscle force could be indirect, via its adipogenic effects or capacity to promote cell differentiation. Based on the present results, this hypothesis should be validated primarily in oxidative muscles since the expression of C/EBPβ was only positively modulated in SOL muscles of rats submitted to HFD.

In response to HFD, intramyocellular lipid accumulation is highly muscle-dependent. Although it is still debatable in mouse models, rat SOL muscles are more prone to accumulate excessive amounts of intramuscular lipids (Shortreed et al., 2009; Kaneko et al., 2011; Campbell et al., 2015; Ciapaite et al., 2015; Komiya et al., 2017). The method used here to measure lipid droplets excluded both intermuscular and myocyte membrane areas. We determined that in rat SOL muscles submitted to HFD, LDs are larger but their number is not increased. Intramyocellular LDs are an important energy substrate pool for β-oxidation in the exercising skeletal muscle, especially in the oxidative type I fibers (van Loon et al., 2003). However, large LDs are not as efficient as smaller droplets for lipid mobilization and turnover, having more of a storage function (Murphy, 2002). Indeed, smaller LDs provide a larger surface area-to-volume ratio, thus favoring lipolysis (Mak, 2012). Intramyocellular lipid accumulation has previously been linked to altered muscle force in mice, independently of mitochondrial oxidative phosphorylation and fiber type (Nunes et al., 2012). The same study hypothesized that large lipid droplets could mechanically impair contraction in the muscle filaments. Further, a mathematical modeling framework simulating intramuscular fat accumulation also suggested mechanical alterations of the muscle as being responsible for diminished force (Rahemi et al., 2015). Indeed, intramuscular lipid could increase the stiffness of the base-material properties affecting fiber shortening and lateral expansion. As mentioned earlier, larger lipid droplets are more prone to induce structural changes leading to these mechanical alterations. Additional characterization of intermyocellular lipid droplets size, number and distribution could add weight to this hypothesis. In the present study, we also observed increased expression of LPL, CD36, DGAT1 and DGAT2. These factors play a critical role in lipid metabolism in the skeletal muscle. For instance, upregulation of LPL could promote the hydrolysis of extracellular triacylglycerol (TAG) into non-esterified fatty acids (NEFA) (Wang and Eckel, 2009). Higher CD36 expression could also stimulate intracellular uptake of NEFA while DGAT1 and DGAT2 would potentiate their re-esterification into TAG as well as their storage in LDs (Jeong et al., 2012). Mediators of intracellular lipid transport, TAG synthesis, LD formation, stabilization and lipolysis appear as attractive targets to clarify the present results and they will require more attention in further studies.

The present results suggest that the effects of HFD on SOL muscle specific force are not mediated through impairments of insulin receptor signaling. This is supported by the observation that the development of insulin resistance and impaired glucose transport are delayed in response to early lipid accumulations in rat skeletal muscle (Bonen et al., 2015). However, the association between intramyocellular lipid accumulation and insulin resistance is well-documented (Phillips et al., 1996; Pan et al., 1997). Hence, it was suggested that LD size in the subsarcolemmal space has a major incidence on the onset of insulin resistance in humans (Nielsen et al., 2017). Also, it was recently suggested that impaired insulin resistance would not occur in response to increased myocellular lipid contents but rather to larger LDs. Other alterations in lipid metabolism, fatty acid oxidation, lipid storage as well as in the activity of LD coat proteins could also have a greater incidence on insulin resistance than absolute lipid levels *per se* (Bosma, 2016). It is likely that young rats display greater metabolic flexibility than older animals and, therefore, a 14-day HFD treatment is too short to induce insulin resistance.

Results presented herein demonstrate that a short-term high fat diet alters specific force in SOL muscles of young rats. This effect cannot be attributed to a remodeling of the muscle fiber phenotype or to impairments in insulin receptor signaling. Further, 2 weeks of HFD induced significant increases in LD size in SOL muscles. This suggests the existence of a causal link between larger LDs and the reduction of specific force in oxidative skeletal muscles. Further studies are needed to clarify the role of distinct lipid species and their trafficking as mediators of LD formation and stabilization, mitochondrial functions, oxidative stress and inflammation in the regulation of skeletal muscle functions in young growing rats.

## Data Availability

The raw data supporting the conclusion of this manuscript will be made available by the authors, without undue reservation, to any qualified researcher.

## Author Contributions

DA contributed to manuscript writing, experiments, data treatment, statistical analyses, tables, and figures. YO, LM, J-PL-G, NA, JM, BS, and LT contributed to experiments and manuscript revision. GG, GD, and A-SC contributed to study design and manuscript revision. DS-P contributed to study design, experiments, and manuscript writing. All authors contributed to manuscript revision and read and approved the submitted version.

## Conflict of Interest Statement

The authors declare that the research was conducted in the absence of any commercial or financial relationships that could be construed as a potential conflict of interest.
