# Supplementary material for: Altered Lipid Metabolism Impairs Skeletal Muscle Force in Young Rats Submitted to a Short-Term High-Fat Diet
Source: Front Physiol. 2018 Sep 26;9:1327. doi: 10.3389/fphys.2018.01327 (PMC6190893; doi:10.3389/fphys.2018.01327)
Supplement: Supplementary file 1 [file Table_1.DOCX]

Supplementary Material

ALTERED LIPID METABOLISM IMPAIRS SKELETAL MUSCLE FORCE IN YOUNG RATS SUBMITTED TO A SHORT-TERM HIGH-FAT DIET

**David E. Andrich, Ya Ou, Lilya Melbouci, Jean-Philippe Leduc-Gaudet, Nickolas Auclair, Jocelyne Mercier, Blandine Secco, Luciane Magri Tomaz, Gilles Gouspillou, Gawiyou Danialou, Alain-Steve Comtois and David H. St-Pierre^*^**

*** Correspondence:**

David H. St-Pierre

Email: [st-pierre.david_h@uqam.ca](mailto:st-pierre.david_h@uqam.ca)

Secondary email: david.stpierre.uqam@gmail.com

**Supplementary Figure 1.** Full western blot showing the expression of insulin signalling proteins IRS-1, total Akt, phospho-Akt, total S6 and phospho-S6 in oxidative (SOL) and glycolytic (EDL) skeletal muscles of young rats submitted to 14 days of HFD or RCD.
