# Supplementary figures and images for: Altered Lipid Metabolism Impairs Skeletal Muscle Force in Young Rats Submitted to a Short-Term High-Fat Diet
Source: Front Physiol. 2018 Sep 26;9:1327. doi: 10.3389/fphys.2018.01327 (PMC6190893; doi:10.3389/fphys.2018.01327)

## Slide 1
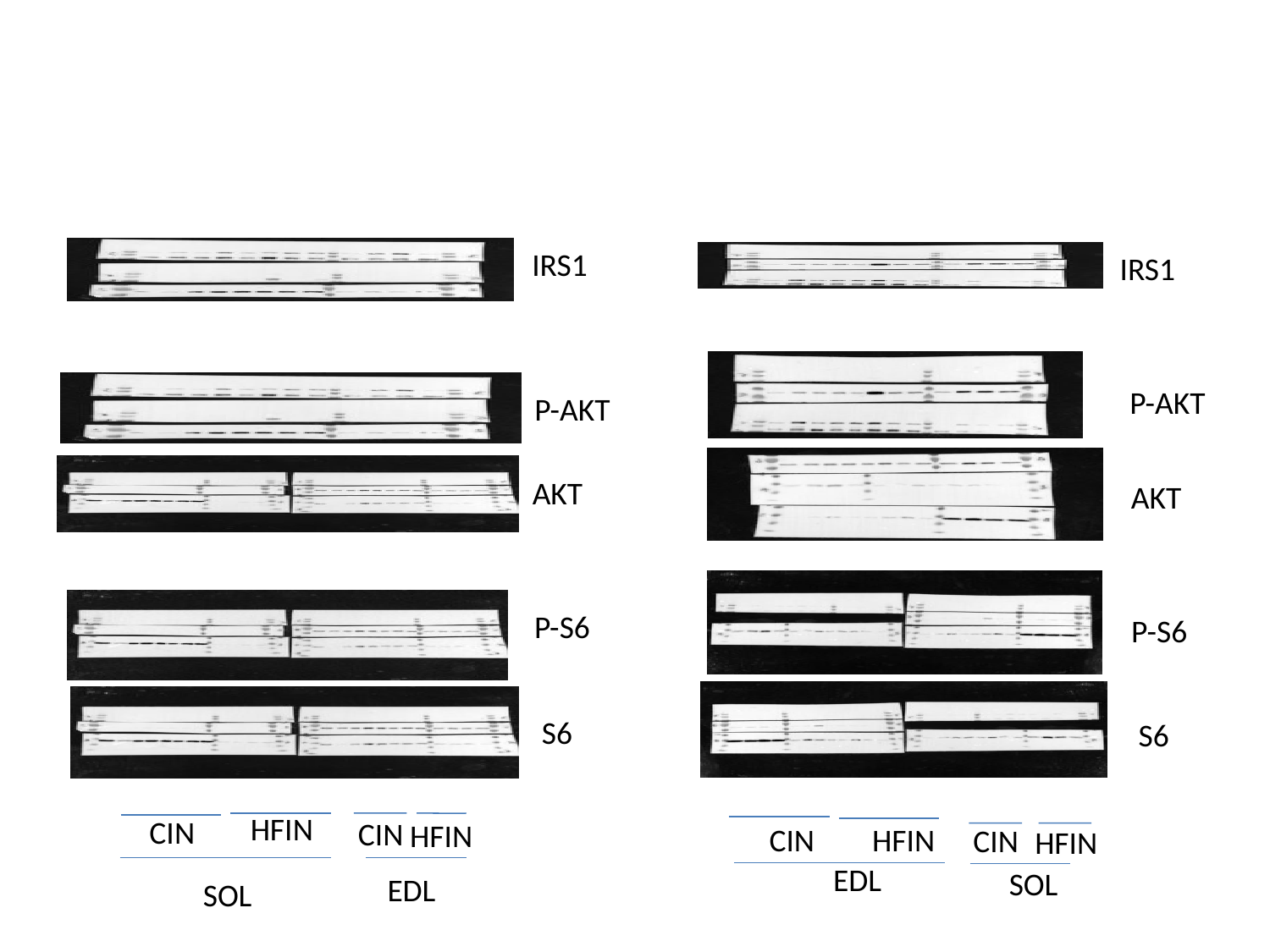

IRS1
IRS1
P-AKT
P-AKT
AKT
AKT
P-S6
P-S6
S6
S6
HFIN
CIN
CIN
HFIN
CIN
HFIN
CIN
HFIN
EDL
SOL
EDL
SOL

Supplement: Supplementary file 2 [file Presentation_1.PPTX]
